# Severe Urban Outdoor Air Pollution and Children’s Structural and Functional Brain Development, From Evidence to Precautionary Strategic Action

**DOI:** 10.3389/fpubh.2018.00095

**Published:** 2018-04-04

**Authors:** Amedeo D’Angiulli

**Affiliations:** ^1^Neuroscience, Carleton University, Ottawa, ON, Canada; ^2^Institute of Interdisciplinary Studies (Child Studies Program), Carleton University, Ottawa, ON, Canada

**Keywords:** outdoor air pollution, urbanization, neurocognitive development, neuroinflammation, precautionary principle, neurodegenerative processes

## Abstract

According to the latest estimates, about 2 billion children around the world are exposed to severe urban outdoor air pollution. Transdisciplinary, multi-method findings from epidemiology, developmental neuroscience, psychology, and pediatrics, show detrimental outcomes associated with pre- and postnatal exposure are found at all ages. Affected brain-related functions include perceptual and sensory information processing, intellectual and cognitive development, memory and executive functions, emotion and self-regulation, and academic achievement. Correspondingly, with the breakdown of natural barriers against entry and translocation of toxic particles in the brain, the most common structural changes are responses promoting neuroinflammation and indicating early neurodegenerative processes. In spite of the gaps in current scientific knowledge and the challenges posed by non-scientific issues that influence policy, the evidence invites the conclusion that urban outdoor air pollution is a serious threat to healthy brain development which may set the conditions for neurodegenerative diseases. Such evidence supports the perspective that urgent strategic precautionary actions, minimizing exposure and attenuating its effects, are needed to protect children and their brain development.

## Air Pollution and Global Child Exposure

Air quality is often defined by indices reflecting concentrations of primary air pollutants: particulate matter (PM), ozone (O_3_), carbon monoxide (CO), sulfur dioxide (SO_2_), nitrogen oxides (NO_x_), and lead (Pb). Most monitoring systems measure PMs, typically, PM_10_ (<10 µm diameter) and PM_2.5_ (<2.5 µm diameter), these and ultrafine PM (UFPM) (<100 µm diameter) are often implicated in brain research. PMs are produced by natural mechanisms and sources and by the chemistry of precursors in the atmosphere; however, they are multiplied by anthropogenic activity: *directly* (e.g., tailpipe and brake emissions from vehicles, residential fuel combustion, power plants, oil refineries, metal processing facilities, etc.) and *indirectly* by altering natural echo systems [i.e., cyclical wildfires emissions (1)]. The anthropogenic surplus is accumulating faster than Earth’s self-regulating mechanisms can sustain (2).

Outdoor air pollution (hereafter *OA-pollution*) is robustly associated with urbanization (3, 4). Global ground-level monitoring of PM_2.5_ in nearly 3,000 cities by the World Health Organization (5) between 2008 and 2015 shows that approximately 98% of cities in low/middle income countries do not meet WHO guideline safety cut-off (annual mean value of PM_2.5_ < 10 μg/m^3^) (5), similarly, in high-income countries 56% of cities are over-limit, and within these, 80% of people are overexposed, with levels ranging between from >10 and >100 µg/m^3^ (10-fold the cut-off). Accordingly, approximately 2 billion exposed children are estimated worldwide [statistics from UNICEF (6) based on Van Donkelaar et al. (7)].

Indoor air pollution is also a major toxic hazard for children. However, children move between indoor and outdoor environments seamlessly, and the many microenvironments between indoor and outdoor are indeterminate. Analysis of children’s exposure in urban environments is much more complex than characterizations of “indoor” and “outdoor,” while children present unique exposure vulnerabilities, in many cases urban pollution incorporates issues implicated by both outdoor and indoor ambient, given their entanglement, raising awareness for urban pollution practically nudges the core overlapping issues.

Between 1990 and 2015, the latest urbanization wave, the estimated indoor pollution-related deaths worldwide remained stable around 2.9 million. However, the estimated OA-pollution-related deaths increased from 2.2 to 4.2 million (8). By 2050, about 70% of the world’s population is forecasted to be urban (9). Consequently, urban OA-pollution seems a more complex, escalating, and pressing risk. Indeed, there is now an extensive literature on brain functions and structure in children exposed to severe chronic urban OA-pollution (hereafter shortened as “exposed children”). In this paper, upon reviewing key transdisciplinary findings (from epidemiology, developmental neuroscience, psychology, and pediatrics), a precautionary perspective is proposed for protecting children’s brains from urban air pollution, despite scientific uncertainty and societal policy issues.

## Effects of Severe Air Pollution on Brain-Related Functions in Children

### Perceptual and Sensory Information Processing

Calderón-Garcidueñas et al. (10) measured brainstem auditory evoked potentials from preschool to adolescence. Compared to controls, exposed children exhibited significant delays in central conduction time of brainstem neural transmission, and auditory, speech, and vestibular performance deficits. Children, teenagers, and young adults from the same community (11) showed more deficits than less-exposed counterparts (OR = 4.03) on the University of Pennsylvania Smell Identification Test—a robust measure of degree of olfactory loss and neurodegenerative progression (12, 13). Although the genetic and cultural variations were likely to be very similar, the effects of sex differences on olfactory performance was a possible confound. The small, cross-sectional convenience samples were another limitation.

### Intellectual and Cognitive Development

Amount of perinatal exposure [e.g., NO_2_, PM, and polycyclic aromatic hydrocarbons (PAHs) in UFPM] is inversely associated with scores in intelligence and intellectual development tests in infants (i.e., first 24 months), preschool, and school children with strongest associations between age 3 and 7. Similarly, cross-sectional and longitudinal studies have shown postnatal protracted exposure is associated with an array of cognitive delays whether measured by IQ scales or similar standardized tests, age 7–18 (14). Many of these studies did not effectively control for key confounds (e.g., associations between lower socioeconomic status and higher OA-pollution residence); nonetheless, findings from both rigorously controlled and weaker studies generally converge.

### Memory and Executive Functions

Severe chronic perinatal and postnatal childhood exposure is associated with significant deficits in tasks measuring working memory, executive and sustained attention, planning and execution of motor or eye-motor coordination, and performance at ages 7–11 (15). The associations between memory/executive functions outcomes and OA-pollution exposure have been confirmed in animal experiments without typical confounds and using ecologically valid exposure levels [e.g., Ref. (16)].

### Emotion and Self-Regulation

An increasing body of evidence is showing associations between traffic-related exposure and diagnoses of psychiatric disorders in children and adolescents (17), self-regulation, and ADHD (18, 19). Although most studies included large, representative samples with clinically relevant community-level results, findings were correlational and most did not control for multiple toxin exposure prenatally or postnatally and moderating factors such as maternal use of psychotropic medications, making it difficult to exclude genetic predispositions. Similarly, the accumulating reported links between prenatal or postnatal exposure and increased risk of autistic spectrum disorders (20) remain difficult to generalize partly because of variation in the pollutant profiles and diagnostic criteria.

## Effects of Severe Air Pollution on Children’s Brain Structure

**Figure 1:**
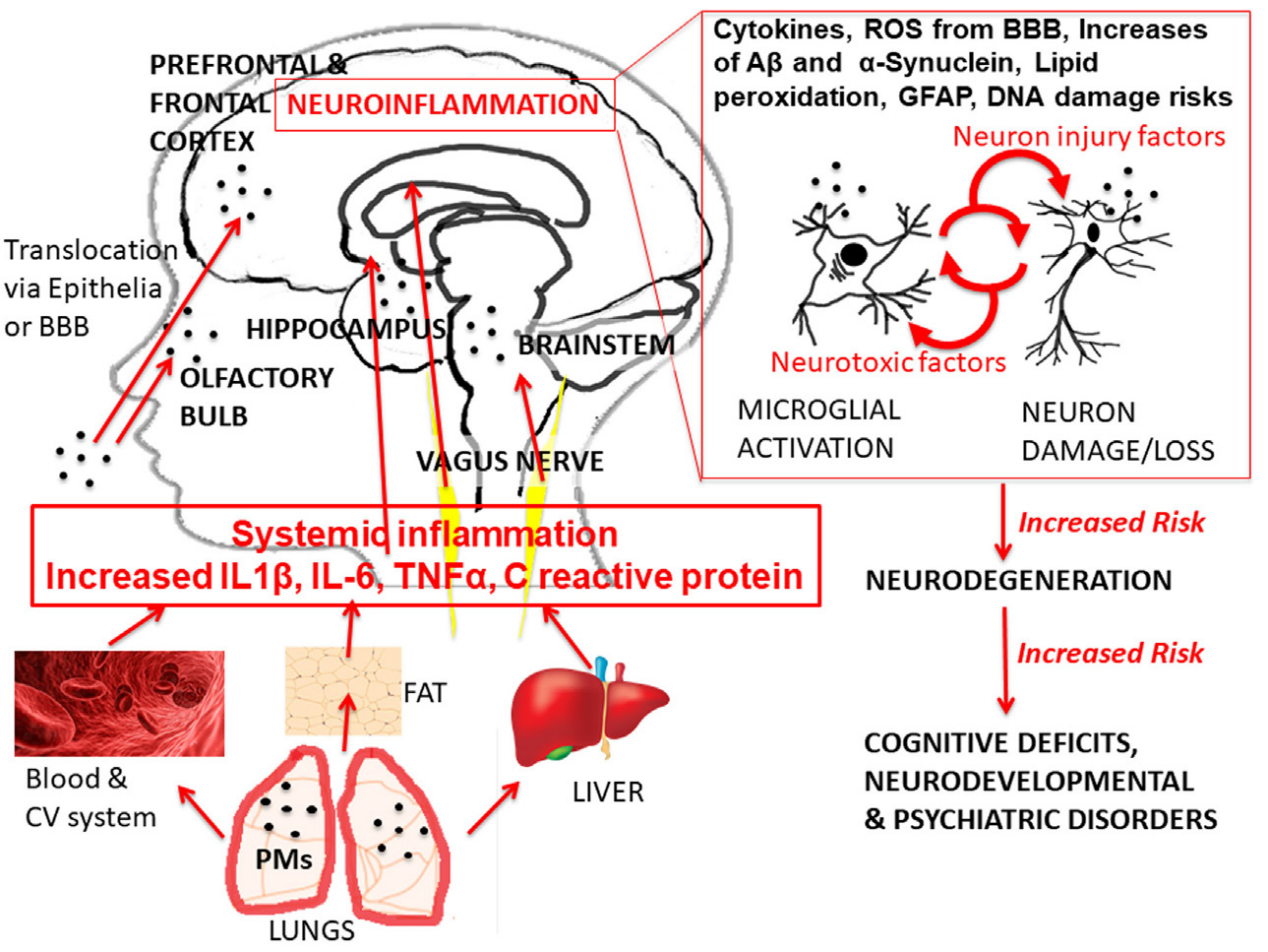
Glance overview of current hypothesized pathways of urban air pollution effects. The graph shows the exposure of urban outdoor air pollution [with specific example of exposure to particulate matter (PM)], the hierarchical cascade, and recursive mechanisms of action leading to the different health effects, with particular focus on healthy brain development outcomes. Abbreviations: IL-6, interleukin 6; IL-1β, interleukin 1 beta; TN F-α, tumor necrosis factor alpha; BBB, brain–blood barrier; ROS, reactive oxygen species; GFAP, astrogliosis; Aβ, beta amyloids. The anatomical illustrations (blood cells, liver, and adipose tissue) are modified versions of copyright- and attribution-free public domain images downloaded from https://pixabay.com/.

Convergent evidence is reviewed from several cohorts of children in the Mexico City Metropolitan Area (MCMA), where PM_2.5_ is estimated to fluctuate annually between 60 and 99 µg/m^3^. Brain changes found in MCMA are consistent with functional outcomes similar to those reviewed above and reported in cities worldwide. A common theme is the role of neuroinflammation (21), see Figure 1.

Traffic-related OA-pollution has been shown to activate proteins responsible for inflammation in the brain. It has also been linked to break down the blood–brain barrier (BBB), which is responsible for keeping harmful particles and chemicals out of the brain. As pollutants wear down this barrier, harmful particles pass into the brain (22). Damage to barriers, such as the BBB and nasal epithelium has been found in young children (23). Children present unique risks of exposure and vulnerabilities. They consume more air and water per unit of body size compared to adults, and they are more active and spend more time outdoors during traffic peak times, for example, during school recessions (24). Children are further at risk because epithelial barriers are not fully developed. Furthermore, young children tend to play closer to the ground, where PM is found in higher concentrations (24).

The body’s innate immune response is turned on when air pollutants enter the body. Cytokines and chemokines and reactive oxygen species (ROS) signal the body to begin an inflammatory immune response. When these immune-inflammatory responses occur chronically or too often, they may lead to widespread loss of brain tissue, indeed this is currently accepted as a necessary, but not sufficient “*deterministic”* [i.e., not *one-to-one cause-effect* (25)] precondition for neurodegeneration (26). Altered ROS were found more frequently in exposed children (56% as compared to 8% in the matched-controls; OR = 17.33) in the prefrontal and frontal areas and associated with deficits in crystallized and fluid cognition (27). Further, they have been associated with evidence of damage to memory centers, in particular, the hippocampus (23) and the olfactory bulb. Neuropathology studies in school-aged children suffering accidental death suggest the olfactory deficits reviewed earlier could be associated with olfactory bulb inflammation. Notably, UFPM was found in olfactory bulb endothelial cytoplasm and basement membranes, and its deposits were associated with other alterations of the auditory brainstem nuclei related to auditory and speech impairments, specifically, the medial superior olive neuronal cell bodies—a finding confirmed in co-resident sudden dead young dogs (28).

Inflammatory protein markers in the brain—interleukins 6 (Il-6) and 1 beta (IL-1β) and tumor necrosis factor alpha (TNF-α)—may readily pass through the BBB. Once past, the BBB can interact with the brain by activating pro-inflammatory mediators that in turn promote alteration of the surrounding brain tissue. This process is followed by a release of cytotoxic species in the brain leading to further inflammatory signaling. Ultimately, this leads to a prolonged inflammatory response in the brain and tissue damage. MRI studies show that exposed children suffer damage to the neurovascular units resulting in neurovascular dysfunction (22). Furthermore, white matter hyperintensities (WMH), characterized by white matter perivascular damage, also associated with global cognitive deficits (29), have been linked to inflammatory protein expression in youth (30, 31). Although WMH are identified by visual inspection, clinical experts’ ratings (as in the studies cited) have good reliability and correlate with MRI measurements (32). Thus, findings suggested OA-pollution damaged key interactive networks of vascular, glial, and neuronal cells; it also increased levels of cytokines. The changes were concurrent to cognitive deficits, specifically, performance on IQ subtests, verbal, and nonverbal (30, 31, 33, 34). The deficits remained even when accounting for differences in household incomes, gender, age, and mother’s IQ (33, 34). MRI volume measurements showed differences in frontal, temporal, and parietal areas between MCMA children and minimally exposed matches (30, 31). Exposure classification was based on community level rather than actual individual-level measurement. However, children were from homogeneous cohorts in both communities (similar socioeconomic backgrounds, same neighborhood, and school) with intergroup data fairly homoscedastic. Therefore, exposure misclassification might have biased effect sizes, but not invalidated the consistent patterns of differences.

Finally, evidence of proteins linked to Alzheimer’s disease (AD) was found in exposed children’s brain. These abnormal proteins are hyperphosphorylated tau (associated with neurofibrillary tangles) and beta amyloid plaques (Aβ1–42) (35). Autopsies from all-life MAMC-resident children succumbing accidental deaths not due to a genetic or traumatic neurodeficit showed both the tangles (14 out of 35, 40%) and plaques (18 out of 35, 51%) in their frontal cortex, whereas none were found in minimally exposed matches (0 out of 8) (30, 31); accidental children’s death is relatively rare in the control cities but, for its 21 million population, proportionally more so in MAMC (36). Because deaths were unrelated to brain, it is extremely improbable that the significant neuropathology findings in MAMC were by-chance false positives and the control null findings were by-chance false negatives. Furthermore, the number of tau tangles and plaques were significantly higher in children carrying the APOEε4 gene, rather than the more common APOEε3 (30, 31). The APOEε4 gene is a well-known genetic risk factor which could put young carriers at risk of developing early-onset AD (35).

## Knowledge Limitations and Future Research

Limitations in developmental OA-pollution research involve the *incompleteness* of findings [(37); see Table 1], scientific *uncertainty* regarding risk assessment [(38); see Table 2], and inconsistent research on outcomes of exposure [(18); see Table 3]. Table 1 summarizes the recommendations of the National Institute of Environmental Health Sciences/National Institute of Health panel (37) to fill knowledge gaps, with main priorities to understand the OA-pollution characteristics necessary to elicit detrimental pro-inflammatory responses, confirm the role of neuroinflammation in toxicity, identify all critical mechanisms, cell types, direct and indirect pathways, and their complex interactions (see Figure 1).

**Table 1:** A selection of future research priorities identified by the National Institute of Environmental Health Sciences/National Institute of Health panel.

Addressing critical research and knowledge gaps
Investigate mechanisms of pollutant entry, distribution, and elimination in the brain. Further investigation is needed to determine the influence of particle size and composition on transport and elimination from the brain. Assessing whether specific chemical (e.g., metals, PAHs) and/or physical properties of PM (e.g., size: UF, PM_2.5_, and PM_10_) are responsible for the inflammatory/neurotoxic effects in the brain and CNS. Identifying populations (aged, young, genotype, low socioeconomic status, high stress, and ongoing CNS disease) that are vulnerable to air pollution using animal and epidemiology studies. Exploration of specific air pollution components and increased risk of neurodevelopmental, neurodegenerative, and mental disorders in humans. Evaluate whether CNS effects occur downstream or independently from cardiovascular or cerebrovascular damage. Utilization of refined exposure estimates to examine long-term air pollution effects on the brain and to elucidate relevant windows of exposure. Adding air pollution exposure component to existing longitudinal pollution cohort studies allows for resource efficient examination of CNS effects. Investigate CNS effects of acute air pollutant exposure during reported peak ozone or particulate matter periods to define temporal resolution. Study air pollutant effects on sensitive subgroups, such as genetically susceptible populations, to highlight mechanisms of importance. Evaluation of subclinical outcomes. Animal studies and MRI neuroimaging technologies to study subclinical white matter disease or infarcts, illuminating underlying disease processes.

*Adapted from Block et al. (37), pp. 15–16*.

**Table 2:** Recommendations for effective application of the precautionary principle.

The Precautionary Principle, adopted by the UN in 1992, states: “In order to protect the environment, the precautionary approach shall be widely applied by states according to their capabilities. Where there are threats of serious or irreversible damage, lack of full scientific certainty shall not be used as a reason for postponing cost-effective measures to prevent environmental degradation.” Proper implementation must consider the following: Preventative action must be used in cases of uncertainty when harm may result. Failing to link harm to a pollutant must be tested when the pollutant is released into public space. The risk of false negative must be reasonable adjusted (*p* = 0.05, from *p* = 0.2) in order to assess pollutant risk before implementation of a technology or policy. The burden to prove the safety of an activity must be the responsibility of the proponents of said activity. A safe activity or technology should demonstrate a *p* = 0.05 false negative error or lower, and a *p* = 0.20 false positive error. The proponents must shoulder the cost and responsibility of the necessary scientific analysis in these cases. A wide range of alternatives should be considered before the implementation of potentially harmful technologies, activities, or policies. Increasing and encouraging the public’s participation in the decision process with leaders of industry. A public panel representing the community should meet and negotiate with industry to asses new industrial and technological concerns. Such interactions must represent the community, understand risk assessment, and have adequate power and resources to deal with the industrial sector from a position of equal power. Citizens must vote according to their ethical and health concerns, communicating their concerns with their elected officials.

*Adapted from Moore (38), pp. 230–237*.

**Table 3:** Early life pollutant exposure: research limitations and challenges.

A significant challenge when studying pollution dose-exposure is controlling/determining dosage and exposure amount. Although there is a positive dose-response relationship, it remains difficult to determine exact exposure amounts and periods of exposure. Exposure is unlikely to be a single occurrence, and the exposure dosage likely fluctuates based on context and environment and remains variable throughout an individual’s lifetime. Studies will often minimally control for moderating factors, such as a parent’s cognition and psychiatric health. It remains difficult to ascribe developmental deficits to *in utero* exposure versus those that occur due to genetic predisposition to psychopathology. There is a lack of standardized neuropsychological, psychosocial, and academic measures across studies. There is a gap in the literature due to a failure to continue recording observations of children past 6 years of age. Long-term developmental and cognitive deficits remain overlooked—a significant oversight considering the greater cognitive and social demand required by the school setting. There remains a need for a practical screening method that pediatricians can use to trigger referrals and early identification. Further research is needed to determine the impact of pollutant exposure on academic skills and the effectiveness of school-based intervention strategies and/or accommodations.

*Adapted from Sullivan and Riccio (39), pp. 187–188*.

The complexity of the underlying mechanisms is a major challenge (40). Pro-inflammatory responses should not be considered simply as single mutually exclusive causes, but rather part of a cascade system of interacting and hierarchical recursive mechanisms that may reflect the result of time-sensitive suppressed protective/repair processes (41, 42).

Such complexity may explain the likelihood of a proportion of immuno-resistant individuals, but also why another proportion of apparently healthy children may only show subtle, preclinical short-term effects. The findings of MAMC young adults showing early signs of neurodegeneration may very well indicate a wear off of the neuroprotection/repair in place during childhood.

And yet, in another proportion of children, effects may go undetected. If participants appear “clinically” healthy, no obvious deficits or complaints would prompt examination, and standard clinical methods may not uncover adverse effects. As possible solutions, neuropsychological testing should include analysis of specific tasks not just global scales, such as IQ quotients, with possibly concurrent functional neuroimaging, structural MRI should capture the whole brain with no gaps, and confounded pathology in diagnostic MR images should be disambiguated functionally.

Future studies may need to address these uncertainties with models that account for the multi-level complex relationships between timing of OA-pollution exposure, neuroinflammation, and neurodevelopment. Moreover, forthcoming efforts should include better understanding of how the brain interacts with the immune system. For instance, DNA methylation status in MAPK pathway genes (43) and telomere length (44) are highly associated with OA-pollution, after adjusting for confounders.

## Policy Implications: Precautionary Action Despite Incomplete Knowledge

It is debatable whether intervention aiming at protecting or improving children’s lives should be delayed or not implemented until scientific knowledge reaches completeness and certainty. The very essence of what we deem “acceptable” harm for children who cannot protect themselves is a topic of study for science, and scientific findings can help defining and refining it; however, ultimately, it pertains ethics and moral values (38).

Although, for some aspects (discussed earlier) it is helpful to investigate the detailed and specific effects of single pollutants, this may have limited practical validity, because many pollutants are highly linked, and it is difficult to separate their effects. There are also considerable interactions between pollutants, which makes it difficult to determine the cause and effect of a single pollutant. Furthermore, burning of biomass fuels for heating, lighting, and cooking, for example, is a major source of OA-pollution in many cities as these activities also occur outside. Concentrations and types of pollutants vary considerably by time and location, depending on cooking schedules and other daily activities, including working hours and transportation rush hours. Hence, a chemical-by-chemical and source-specific assessment of risk does not reflect the cumulative impacts of multiple toxic stresses posed by severe OA-pollution (45).

Thus, following an urban environmental justice perspective (46), risk assessment cannot be narrowly focused on calculating the probability or significant effects of acceptable impact of independent single pollutants from single sources and through single exposure pathways, since the underlying (untested) assumption is that children’s brains can tolerate an endless accumulation of single “acceptable” insults (47).

An alternative concept is that the developmental effect of extreme chronic urban OA-pollution can be defined as total cumulative serious harm to brain development resulting from involuntary exposure to multiple hazardous or toxic pollutants over time, with serious threats of developing neurodegenerative disease in a clinically important fraction of children’s population.

The above concept has an important implication: the real health cost of OA-pollution is incalculable, as is the total economic profit of polluting; commensurably, the latter would include not only industrial production, but also our own everyday behaviors, such as, for example, using transportation vehicles to do just about anything. Therefore, given its fundamental indeterminacy, cost/benefit analysis offers modest validity for policy or decision making.

Benefit/cost analysis is only part of what determines the basis of policy and intervention and is not the only or even the chief justification, the most important rationale for both are ethical and moral values, such as protecting children who are involuntarily exposed. Proposing an application of the *precautionary principle* [(48); see Table 2], Moore (38) outlined ethical and rational justifications for urgent strategic actions to attenuate and minimize children’s exposures despite incomplete scientific knowledge. Two examples of viable strategic precautionary actions against OA-pollution are discussed next.

A possible precautionary strategy is translational, involving experts from different fields working together to define and communicate appropriately the threats of OA-pollution to families, the public, policy makers, politicians, and stakeholders (21). This involves increasing awareness of times, places, and circumstances in which children become most exposed and how to reduce and minimize such exposure, namely, awareness of stationary polluting sources and major roadways, monitoring of air quality, of activities, such as cooking, and improving ventilation (39).

A second strategy is continuous identification and monitoring of the associations between exposure and children’s brain health (6). Consequently, neuropsychological and psychoeducational screening and testing for early identification and intervention could target subgroups of children who are at higher risk [i.e., attending daycares or schools near major roads (18, 49)]. Various appropriate assessment methods may be necessary for identification and possibly follow-up to monitor progress and rate of development in response to continued exposure (39). In some cases, more in-depth targeted investigations should involve combined functional neuroimaging technologies (i.e., EEG/ERP and fMRI).

## Conclusion

In conclusion, urban OA-pollution poses a serious threat to healthy brain development, with functional and structural changes linked with neurodegeneration (50). Dose-response and threshold approaches in practice lag behind the specific complexity of chronic urban exposure, the threshold for compromised neurocognitive development may be surpassed at birth already (44, 51) and, as exemplified by airborne Pb regulatory controversies, the acceptable pediatric dosage is problematic even at low-level exposures (52). The present perspective argued for precautionary researchers/practitioners based actions; these are “strategic” because they need to work around the polluting activities that are now deemed necessary for modern life and economy. As OA-pollution and urbanization continue to rise globally (53), precautionary actions to minimize and attenuate children’s exposure become not only a priority, but also an emergency.

## Author Contributions

AD designed, drafted, revised and finalized drafts and manuscript.

## Conflict of Interest Statement

The authors declare that the research was conducted in the absence of any commercial or financial relationships that could be construed as a potential conflict of interest.
